# Blunted blades: new CRISPR-derived technologies to dissect microbial multi-drug resistance and biofilm formation

**DOI:** 10.1128/msphere.00642-23

**Published:** 2024-03-21

**Authors:** Christopher Gager, Ana L. Flores-Mireles

**Affiliations:** 1 Department of Biological Sciences, University of Notre Dame, Notre Dame, Indiana, USA; 2 W. M. Keck Center for Transgene Research, University of Notre Dame, Notre Dame, Indiana, USA; University of Georgia, Athens, Georgia, USA

**Keywords:** antimicrobial, CRISPRi, fungi, bacteria, multi-drug resistance, biofilms, essential genes, CRISPRa, *E. coli*, mycobacterium, *Candida*, pathogens

## Abstract

The spread of multi-drug-resistant (MDR) pathogens has rapidly outpaced the development of effective treatments. Diverse resistance mechanisms further limit the effectiveness of our best treatments, including multi-drug regimens and last line-of-defense antimicrobials. Biofilm formation is a powerful component of microbial pathogenesis, providing a scaffold for efficient colonization and shielding against anti-microbials, which further complicates drug resistance studies. Early genetic knockout tools didn’t allow the study of essential genes, but clustered regularly interspaced palindromic repeat inference (CRISPRi) technologies have overcome this challenge via genetic silencing. These tools rapidly evolved to meet new demands and exploit native CRISPR systems. Modern tools range from the creation of massive CRISPRi libraries to tunable modulation of gene expression with CRISPR activation (CRISPRa). This review discusses the rapid expansion of CRISPRi/a-based technologies, their use in investigating MDR and biofilm formation, and how this drives further development of a potent tool to comprehensively examine multi-drug resistance.

## INTRODUCTION

Microbial infections and rapid development of antimicrobial resistance are major threats to global health (1, 2). Treatment options for multi-drug-resistant (MDR) bacterial and fungal pathogens are becoming limited and pose a major burden in hospital and outpatient settings (3). Another contributing factor to MDR is biofilm formation, which protects pathogenic communities from antimicrobial treatments and the immune response, allowing persistent colonization and chronic infections (4–8). MDR development and biofilm formation are complex processes associated with essential and non-essential pathways (9). Transposon (Tn) mutagenesis technologies have helped to uncover non-essential genes involved in these pathways, but essential gene contributions remained unclear. The CRISPRi technology overcame this limitation, allowing knockdown of essential genes to dissect their roles in microbial behaviors including antibiotic resistance, biofilm formation, virulence, and host-microbe interactions. Importantly, this technology has enabled the discovery of new antimicrobial and biofilm targets that otherwise were not possible.

CRISPRi was adapted from native CRISPR systems in bacteria, which play a role in adaptive immunity for prokaryotes (10). Broadly, CRISPRs work by incorporating sequences of foreign DNA as spacers between “palindromic repeats” in a CRISPR loci. These spacers are analogous to sgRNA and are a component of mature CRISPR-associated RNA (crRNA). After post-transcriptional processing, mature crRNA complexes with nucleases and targets the complementary sequence to the spacer. This allows the overall system to bind and degrade previously encountered foreign nucleic acids (11). This system was quickly adopted into many genetic toolkits, initially using *Streptococcus pyogenes* Cas9 nuclease to cause a double-stranded cut at the target location. These are repaired by error-prone non-homologous end joining (NHEJ) in eukaryotes, usually disrupting the gene, also known as “knocking out” (KO). While many prokaryotes lack this mechanism of repair, CRISPR-KO through NHEJ is still possible (12, 13). Designing synthetic single guide RNA (sgRNA) allows the targeting of nearly any location in a genome, though the site needs to be next to specific nucleotide sequences known as protospacer adjacent motifs (PAM sites) (14–16).

The first CRISPRi systems incorporated the same “recognize target sequence and guide nuclease for subsequent degradation” design as baseline CRISPR, but with a catalytically dead Cas9 variant (dCas9) (16–19). Instead of making double-stranded cuts, mutations in both of the Cas9 catalytic domains effectively dull the “molecular scissors.” Though unable to cut DNA at the sgRNA target, bound dCas9 sterically hinders transcription initiation or elongation. sgRNA design strategies use this inhibition to allow selective knockdown of nearly any gene, including essential genes (20). When CRISPRi systems are combined with inducible promoters, titratable levels of gene repression are possible, as opposed to the binary “present or completely knocked out” phenotype of CRISPR-KO (21). CRISPRi screens can therefore be truly genome wide, as essential genes can be investigated through tunable knockdown.

**Fig 1:**
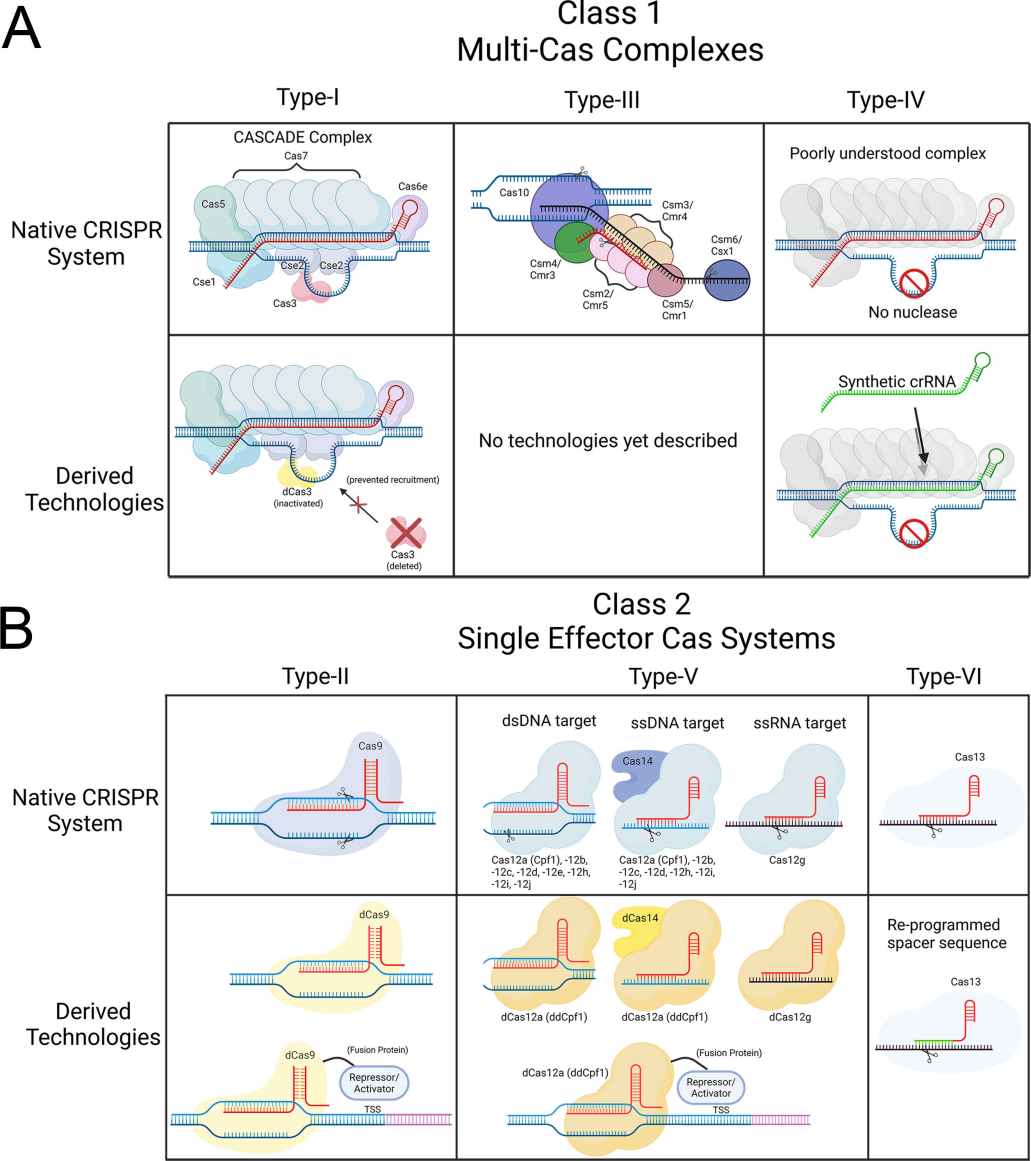
Native CRISPR systems and their derived technologies. CRISPR systems are diverse but can be sorted into two classes. Class 1 systems (**A**) involve multi-Cas complexes whereas class 2 systems (**B**) require a single nuclease. There are three types of systems in each class, and all but one has been utilized in CRISPRi technology. Cas nuclease and complexes are guided by crRNA (red) to either host DNA (blue) or host RNA, usually mRNA transcripts (black). Figure was created using BioRender.

CRISPRi built around dCas9 has been further modified through the creation of dCas9 fusion proteins. CRISPRi using dCas9 fused to transcription repressors provides enhanced gene knockdown in some tools (Fig. 1B) (22, 23). CRISPR activation (CRISPRa) systems use sgRNA to guide dCas9 fused to transcriptional activators to intended sites, typically upstream of the target gene. Instead of physically blocking transcription, dCas9 positions the fused transcriptional activator near features such as promoter sequences to induce gene expression (Fig. 1B) (19, 24). CRISPRa was described as early as 2013, where dCas9 fused with the omega subunit of RNA polymerase led to targeted gene activation in *Escherichia coli* (19). More recent technologies have expanded this technique to fungal pathogens, utilizing different transcriptional activators (24).

It is important to note that not all CRISPR-derived tools are built around dCas9. CRISPR systems are quite diverse and are categorized as class 1 or class 2, which are further divided into Types I through VI. Class 1 multiple-Cas systems consist of Type I, III, and IV systems, while class 2 systems utilize a single effector Cas protein and include Types II, V, and VI (25, 26). An overview of the native mechanisms and the technology derived from them can be seen in Fig. 1. Therefore, CRISPRi/a that utilizes dCas9 is built off a Type II CRISPR system. Its simplicity and well-described nature led to the creation of gene modulation tools in Gram-negative, indeterminate, positive, and fungal species (15, 19, 22, 27, 28).

Class 2 CRISPR also includes Types V and VI. Type V usually utilizes Cas12 or Cas14 (Fig. 1B). Cas14 is unique by only cutting single-stranded DNA (ssDNA) instead of double-stranded DNA (dsDNA) and does not require a PAM site (29). There are many Cas12 subtypes, with most able to target both ssDNA and dsDNA (30). A distinct subtype, Cas12g, only cleaves RNA substrates (30). Among these nucleases, deactivated Cas12a (Cpf1) has already been used to generate CRISPRi tools (Fig. 1B) (31, 32). Type VI CRISPR utilizes the RNA-specific nuclease Cas13, and like the class 1 Type IV system, this functions as native CRISPRi (Fig. 1B) (33).

Despite the frequency of class 2 systems in CRISPRi tools, native class 1 systems account for ~90% of identified CRISPR loci (34). This includes the variety of Type I subsystems, Type IA through 1F, many with additional subvariants. These exhibit different arrangements of various Cas proteins, but all feature Cascade, which are multi-Cas complexes that recruit an effector nuclease for DNA degradation (25). Type III systems utilize a complex of Cas10 proteins similar to the Cascade seen in Type I (25). Type III systems target both DNA and RNA, including the crRNA (35). This self-targeting mechanism has likely hindered Type III CRISPRi development (Fig. 1A) (35, 36). Interestingly, Type IV CRISPR systems lack a functional nuclease entirely, and therefore, the Cas complex only sterically hinders transcription, essentially working as a native CRISPRi system (Fig. 1A) (37).

All but Type III systems have been adapted to create CRISPRi technologies, which have continued to evolve as they are leveraged to study pathogenesis across a multitude of species (37–40). CRISPRa has been developed alongside the earliest CRISPRi tools and has been successfully utilized in some pathogens. Therefore, this review will discuss how the versatility of CRISPRi/a technologies has aided in the identification and validation of conditionally essential genes within pathogenic species that promote drug resistance and biofilm formation (Table 1) as well as how these studies have contributed to the expansion of available CRISPRi-based tools.

**TABLE 1:** CRISPRi/a applications by species

Pathogen	CRISPR type	Target(s) of interest	Mechanism affected	Reference
Gram-negative bacteria				
*Escherichia coli*	Type II CRISPRi inducible	*intI1*, *sul1*, *dfrB2* cassette	Resistance gene acquisition, drug resistance	(41)
	Type II CRISPRi inducible	*acrA*, *acrB*, *tolC*	Drug resistance, biofilm formation	(42)
	Type II CRISPRi inducible	*bla*_KPC-2_	Drug resistance	(43)
	Type II CRISPRi inducible	*luxS*	Biofilm formation	(27)
	Type II CRISPRi inducible	Genome wide	sgRNA design	(44)
*Pseudomonas* spp.	Type I CRISPRi integrative	*CzcR*, *mexA*, *mexG*	Drug resistance	(38)
	Repurposed native Type I CRISPRi	*mexB*, *mexF*, *mexH*	Drug resistance	(45)
	Type II CRISPRi inducible	*PA0715*	Drug resistance, biofilm formation	(46)
	Type II CRISPRi integrative, inducible	*gacS*, *dipA*, *bifA*, *rimA*	Biofilm formation	(47)
	Type II CRISPRi	*exsA*	*In vivo* virulence	(48)
*Klebsiella pneumoniae*	Type II CRISPRi integrative plasmid	*bla*_NDM-1_, *bla*_SHV-12_	Drug resistance	(43)
	Type II CRISPRi integrative plasmid	*folA*	Drug resistance	(49)
	Type II CRISPRi-Seq integrative plasmid	*folB*, *folP*, *waaE*, *fldA*, *pal*, *yciS*, *rib*	Drug resistance, *in vivo* virulence	(50)
*Acinetobacter baumannii*	Type II CRISPRi integrative, inducible	*ftsI*, *murA*, *dapA*, *murJ*	Drug resistance	(51)
Gram-indeterminate bacteria				
*Mycobacterium* spp.	Type II CRISPRi integrative, inducible	*mmpSL5*, *rv0678*, *atpC*, *atpB*, *atpH*, *pks13*, *topA*	Drug resistance	(52)
	Type II CRISPRi integrative, inducible	Genome wide, *Def*, Clp complex	Drug resistance	(28)
	Type II CRISPRi integrative, inducible	*mtrA*, *whiB7*, *ettA*	Drug resistance	(53)
	Type II CRISPRi integrative, inducible	*namH*, *murT*, *gatD*	Drug resistance, immune evasion	(54)
Gram-positive bacteria				
*Enterococcus faecalis*	Type II CRISPRi inducible	*croR*, *ebpA*, *ebpABC* operon	Drug resistance, biofilm formation, immune evasion	(15)
	Type II CRISPRi integrative, inducible	*rv3645*	Drug resistance	(55)
	Type II CRISPRi integrative, inducible	*whiB7*, *Bla*, *ftsZ*, *topA*	Drug resistance	(56)
*Clostridium difficile*	Type II CRISPRi inducible	*pbp-0712*	Drug resistance	(21)
	Type II CRISPRi inducible	*HexSDF*	Drug resistance	(57)
*Staphylococcus* spp.	Type II CRISPRi inducible	*hla*, *spa*, *mecA*	Drug resistance	(58)
	Type II CRISPRi inducible	*blaZ*	Drug resistance	(59)
	Type II CRISPRi sgRNA construction	Genome wide, *ctaABM*, *mnhABCDEFG*, *ndh*, *mvaD*, *mvak2*, *topA*	sgRNA design, drug resistance	(60)
	Type II CRISPRi inducible	*vigR* 3′UTR, *isaA*	Drug resistance	(61)
	Type II CRISPRi inducible	*dltABCD*, *pbp4*, *vraFG*, *ezrA*, *rpsF-ssb-rpsR*, *nrdF*, *kapB*, *sagB*, *aroB*, *unfA*	Drug resistance	(20)
*Streptococcus pneumoniae*	Type II CRISPRi-Seq inducible	*purA*, *metK*	*In vivo* virulence	(62)
Fungal				
*Saccharomyces cerevisiae*	Type II CRISPRi inducible	*ERG25*	Drug resistance	(23)
*Candida albicans*	Type II CRISPRi integrative	*ADE2*	Drug resistance	(22)
	Type II CRISPRi/a inducible	*ALS3*, *YAK1*, *CDR1*, *ALS1*	Drug resistance, biofilm formation, *in vivo* virulence	(24, 63)
*Nakaseomyces glabrata*	Type II CRISPRi inducible	*ALG3*, *URA3*	Drug resistance	(64)
	Type II CRISPRa	*PDR1*, *STE11*, *SLT2*, *EFG1*	Drug resistance, biofilm formation	(65)
*Penicillium rubens*	Type II CRISPRa	*macR*	Antimicrobial production	(66)
*Aspergillus niger*	Type II CRISPRi inducible	*DAC1*	Spore-like propagule formation (drug resistance)	(67)
*Magnaporthe oryzae*	Type II CRISPRi	*MoATG3*	Virulence factor	(68)
*Ustilaginoidea virens*	Type II CRISPRi	*UvPal1*	Virulence factor	(68)

## MULTI-DRUG RESISTANCE

Both bacteria and fungi show great plasticity in surviving antimicrobial treatments. Importantly, MDR organisms have outcompeted our capacity to produce new antibiotics or therapies to treat infections, especially the clinically relevant ESKAPE pathogens (*Enterococcus* spp., *Staphylococcus aureus*, *Klebsiella pneumoniae*, *Acinetobacter baumannii*, *Pseudomonas aeruginosa*, and *Enterobacter* spp.) (69). Therefore, understanding the role of essential pathways involved in antimicrobial resistance is crucial to develop efficient therapies against Gram-negative, indeterminate, and positive bacterial and fungal species.

## GRAM-NEGATIVE BACTERIA

### Escherichia coli

*Escherichia coli* is among the most common nosocomial pathogens, especially in urinary tract infections. These bacteria often possess antibiotic resistance via β-lactamases, acquired though horizontal gene transfer (HGT). This has led to additional carbapenem resistance among some nosocomial *E. coli* strains (70). Acquisition of drug resistance genes from a diverse range of pathogenic bacteria can be attributable to mobile genetic elements, including Class 1 integrons (71). Due to the genetic mobility and diversity of carried resistance genes, Class 1 integrons are difficult to target for elimination. Utilization of CRISPRi has proven successful in inhibiting them in *E. coli*, decreasing the expression of integrase (*intI1*) by ~96% which resulted in 1,000-fold reduction in HGT rate (41). Furthermore, multiple sgRNAs were designed to target the *dfrB2* cassette and *sul1*, which encode a dihydrofolate reductase and a dihydropteroate synthase. These promote resistance to trimethoprim and sulfamethoxazole, respectively (41, 72, 73).

One mechanism of MDR in *E. coli* is to express efflux pumps like AcrAB-TolC. This pump provides resistance to multiple antibiotics, including carbapenems, erythromycin, tetracycline, and rifampicin (74, 75). AcrAB-TolC consists of the outer membrane channel, TolC, which is connected by AcrA to the inner membrane transporter AcrB (76). Using an engineered CRISPRi system, *acrA*, *acrB*, and *tolC* were targeted independently or simultaneously by using plasmids containing clustered sgRNAs. Single guide targeting of *acrA* lead to downregulation of *acrB* and vice versa, while neither reduced *tolC* expression. Given the location of *acrA* and *acrB* within their operon, this was unsurprising. Interestingly, single guides targeting *tolC* did reduce expression of *acrA* and *acrB*. By clustering sgRNA sequences, simultaneous targeting of each gene was achieved. Both single and simultaneous silencing of the efflux pump genes increased susceptibility to rifampicin, erythromycin, and tetracycline, with simultaneous targeting outperforming single sgRNA plasmids (42). These findings indicate that single-target silencing can cause repression in other genes within the studied system, while targeting multiple genes can enhance overall repression.

A potential complication for CRISPRi technology is the presence of multi-copy resistance genes on natural plasmids, especially in clinical strains. The Yao group engineered an all-in-one CRISPRi system to overcome this issue (43). This system was tested in the clinical *E. coli* strain E0171, which contains two copies of the carbapenemase gene *bla_KPC-2_*. Using a single sgRNA that targets the non-template strand of its coding region, they were able to target multi-copy *bla_KPC-2_* to reduce expression 600-fold, making *E. coli* susceptible to meropenem (43).

### Pseudomonas aeruginosa

Development of genetic tools for *P. aeruginosa* has proven difficult, particularly among MDR strains. Some laboratory strains permit the use of Type II dCas9 tools, like PAO1. The unidentified essential gene *PA0715* was characterized in PAO1 using CRISPRi, revealing a role in amino acid, carbohydrate, ketone body, and organic salt metabolic pathways, as well as motility. After *PA0715* silencing, PAO1 was sensitized to sample antibiotics from penicillin, third-generation quinolone, aminoglycoside, and macrolide antibiotics, suggesting it presents a potential new antibiotic target (46).

Genetic tool development is complicated by native Type IF CRISPR systems, which are found in up to 30% of *P. aeruginosa* strains (77). These target foreign DNA, including engineered CRISPRi constructs (45, 77, 78). Xu and collaborators demonstrated that these native systems can be repurposed, using the MDR strain PA154197. After modifying the native CRISPR, they used it to target the efflux pumps MexAB-OprM, MexEF-OprN, and MexGHI-OpmD by knocking out the components *mexB*, *mexF*, and *mexH*, resulting in increased sensitivity to fluoroquinolones and beta-lactams (45). Although this work used CRISPR-KO, it demonstrated successful modification of native CRISPR systems in *P. aeruginosa* clinical isolates (78).

Chen and collaborators created a CRISPRi tool by removing the *cas2-3* gene from Type IF CRISPR that integrates at the *attB* site in the *P. aeruginosa* chromosome (78). This design allows genetic silencing in clinical MDR strains, where intrinsic resistances make selection-based plasmid retention difficult (78). This system is specific to *P. aeruginosa*, and strains with functional native Type IF CRISPRs will eventually target and eliminate the foreign CRISPRi sequence (38). To overcome this challenge, a novel plasmid-based CRISPRi platform (CSYi) was created. To protect against native CRISPR targeting and destruction, they added two anti-CRISPR proteins AcrlF3 and AcrlF23 to the plasmid (38). This system was successfully validated by repressing expression of CzcR, a zinc regulator, by ~50% (38). CzcR modulates multi-drug resistance in *Pseudomonas* through repression of OprD, a porin responsible for carbapenem uptake, and both MexAB-OprM and MexGHI-OpmD efflux pumps (38). CzcR repression sensitized *Pseudomonas* to levofloxacin, proving its role in carbapenem resistance (38). Importantly, this plasmid is not limited to *Pseudomonas*, as it was also shown to work in *E. coli* strains (38).

### Klebsiella pneumoniae

Type I CRISPR loci are prevalent in MDR isolates of the ESKAPE pathogen *K. pneumoniae*, which also frequently harbor multiple resistance genes on large plasmids (79). Through one-step cloning, an inducible CRISPRi system was developed to silence multiple resistance genes in pNK01067-NDM-1, a natural MDR plasmid in a *K. pneumoniae* isolate. Single-target silencing of bla_NDM-1_ reduced meropenem resistance by ~1,000-fold and modifications to the CRISPRi design allowed targeting of both *bla*_NDM-1_ and *bla*_SHV-12_ on the large MDR plasmid, making the isolate susceptible to meropenem and aztreonam. Importantly, gene silencing on the scale of an entire plasmid operon was also possible, by targeting the promoter region of *bla*_NDM-1,_ which silenced the *bla*_NDM-1_-*ble*_MBL_-*trpF* operon (43).

Mobile-CRISPRi in *K. pneumoniae* has been successful. This system uses Type II dCas9 CRISPRi built for ease of library construction, conjugative transfer across species, and stable genomic integration. Using this system, the *K. pneumoniae* essential gene *folA* was targeted. This gene encodes dihydrofolate reductase and is the target of trimethoprim. Complete *folA* gene silencing was lethal, while partial knockdown sensitized *K. pneumoniae* to the drug (49).

This system has also proven useful in a screening strategy similar to Tn-Seq, to assess which genes or pathways have vital roles in antimicrobial resistance. Mobile-CRISPRi-Seq was applied to 870 predicted essential genes under antibiotic pressures at 25% MIC, which identified *folB* and *folP* as trimethoprim resistance genes (50). Additional genes of interest include *waaE*, a component of LPS biosynthesis and polymyxin B resistance, and *fldA*, an electron transport carrier and contributor to beta-lactam resistance (50).

### Acinetobacter baumannii

*A. baumannii* is an emerging MDR nosocomial pathogen, and its treatment is increasingly challenging (80). To understand *A. baumannii* mechanisms of drug resistance, a CRISPRi library was created and screened using last-resort antibiotics including fosfomycin, colistin, imipenem, meropenem, and rifampicin (51). Knockdown of cell wall assembly, peptidoglycan synthesis, and translocation genes (*ftsI*, *murA*, *dapA*, and *murJ*) resulted in increased sensitivity to imipenem and meropenem (51). Interestingly, treatment of *A. baumannii* with fosfomycin—which targets the product of *murA*—was ineffective due to efflux pump activity. However, when fosfomycin and imipenem were administered in tandem, the two exhibited synergistic effects (51). Further antibiotic-gene interaction analysis showed that colistin and rifampicin have strong opposite phenotypes for genes encoding NADH dehydrogenase complex I (NDH-1) and lipooligosaccharide. CRISPRi knockdown of NDH-1 function recapitulated the reduced membrane permeability found in rifampicin resistance. This sensitizes *A. baumannii* to colistin, which inhibits NDH-2. Importantly, these results indicate how anticorrelated phenotypes have been unveiled due to CRISPRi, to better exploit synergistic antibacterial treatment (51).

## GRAM-INDETERMINATE BACTERIA

### *Mycobacterium* spp.

Given the global prevalence and severity of *Mycobacterium tuberculosis* (Mtb), it is not surprising that the CRISPRi technology has been quickly added to the arsenal that aids in understanding the mechanisms of mycobacterial MDR (81). In the Mtb field, CRISPRi is a powerful tool for examining essential genes and has also been effective in conjunction with KO libraries, including CRISPR-KO and Tn-seq libraries (52). When used in conjunction with KO libraries, essential genes identified by CRISPRi can be further explored to determine whether a polar effect contributes to their essentiality (52). Using this strategy, the Sun lab found 29 essential genes that impacted resistance or susceptibility to the antimycobacterial bedaquiline (BDQ), which inhibits the mycobacterial ATP synthase (52). This was achieved by growing both CRISPR-KO and CRISPRi libraries to late-log phase, mimicking *in vivo* mycobacterial growth conditions before adding BDQ (52). While both libraries detected genes resistant and susceptible to BDQ treatment, 29 essential genes were only identifiable by CRISPRi. One discovery included the drug efflux pump component *mmpSL5* (52). The absence of this gene resulted in BDQ sensitivity while inactivation of mmpSL5’s repressor, *rv0678*, led to acquired BDQ resistance by overexpressing the efflux pump (52). Additionally, they found that many essential genes involved in ATP synthesis such as *atpB*, *atpC*, and *atpH* are potential synergistic targets for antimicrobial effects. Another potential synergistic gene only detectable by CRISPRi in this study was *pks13* (52). Pks13 is an essential enzyme that forms mycolic acids and previously was found to be the target of the antimicrobial drug, TAM16 (82). This screen also unveiled unpredicted factors to play a role in drug-gene interactions such as Topoisomerase I (*topA*). The *topA* depletion increased BDQ sensitivity, suggesting that Topoisomerase I may indirectly affect ATP homeostasis (52).

Bosch and colleagues utilized a titratable CRISPRi platform to measure genetic vulnerability in Mtb, including that of a hypervirulent isolate. They constructed a CRISPRi library against 98.2% of all annotated Mtb genes. Instead of inducible sgRNA, this system used Sth1dCas9 instead of dCas9, which allows for a gradient of gene knockdown through recognition of non-canonical PAM sites. The estimated sgRNA strength was then used to predict the degree of gene knockdown, which was used in conjunction with the observed fitness cost in the final model of genetic vulnerability. This process was validated in *Mycobacterium smegmatis* and resulted in a quantitative vulnerability index for ~93% of all TnSeq essential Mtb genes (28). Pathway enrichment of the most vulnerable genes revealed protein synthesis and tRNA synthesis were vulnerable targets, while amino acid biosynthesis was not. The Clp protease complex was found among the most vulnerable gene set, validating active interest in antimycobacterials targeting the protease (28, 83). In contrast, peptide deformalyse *def* is an essential Mtb gene previously suggested as a potential antimycobacterial target (84). The genome-wide CRISPRi assessment revealed *def* as highly invulnerable, suggesting that some essential genes are not good candidates for successful drug development (28).

A CRISPRi chemical genetics platform has been used to titer the expression of Mtb genes (53). By utilizing this approach with multiple antimicrobials, Li and collaborators uncovered multiple mechanisms of acquired resistance, including the response regulator *mtrA*. Knockdown of *mtrA* sensitized Mtb to rifampicin, vancomycin, and BDQ and impaired intracellular survival within macrophages (53). Ethidium bromide and a fluorescent vancomycin conjugate both showed that *mtrA* knockdown leads to increased cell envelope permeability (53).

Tuberculosis antibiotic regimens do not include the use of beta-lactams; however, a combinatory therapy with β-lactamase inhibitors is a prospective strategy to treat MDR Mtb (54). To dissect whether peptidoglycan modifications could promote synergistic effects with antimicrobial resistance and intracellular survival, Silveiro and collaborators CRISPRi silenced *namH* and *murT/gatD*. These encode enzymes responsible for the peptidoglycan modifications D-*iso*-glutamate amidation and *N*-glycosylation of muramic acid, respectively (54). By using CRISPRi silencing in *M. smegmatis* strains, which lack the β-lactamase (*bla*S), they found that *namH* was essential to mycobacterial survival while *murT*/*gatD* was dispensable. Furthermore, silencing of *namH* affected cefotaxime and isoniazid resistance and silencing *murT/gatD* reduced resistance to β-lactams. Furthermore, simultaneous depletion of both genes not only resulted in synergistic increasing of beta-lactam susceptibility but also significantly promoted killing by macrophages (54). As these modifications were highly conserved in a set of 172 clinical tuberculosis strains, these results suggest that peptidoglycan modifications contribute to pathogenicity in Mtb and could be a potential therapeutic target (54).

Recent studies have shown that Mtb dedicates great effort to produce, sense, and degrade cyclic AMP (cAMP) (55, 85–87), suggesting that this molecule may play a role in Mtb physiology. Interestingly, there is only one Mtb-essential adenylate cyclase (*rv3645*) that catalyzes the conversion of ATP to cAMP (55). To dissect the potential roles of cAMP in Mtb, this adenylate cyclase was silenced by CRISPRi, showing that the lack of cAMP increased Mtb sensitivity to vancomycin, rifampicin, and clarithromycin (55). Furthermore, cAMP reduction inhibited Mtb growth in the presence of long-chain fatty acids, a host-relevant carbon source. This study’s finding that cAMP signaling is important for MDR and fatty acid metabolism suggests that its disruption may provide another avenue for Mtb management (55).

CRISPRi chemical genetics identified that loss of function of the essential gene *rv2477c* in Mtb confers resistance to streptomycin, amikacin, ethambutol, rifampicin, and levoflozacin (53). This gene is an ortholog of the *E. coli* gene *ettA*, which is involved in the translation elongation cycle; however, it is not essential in *E. coli* (88, 89). Interestingly, silencing of the *ettA* homolog in *M. smegmatis* resulted in upregulation of two proteins, HflX and Eis, which are part of the *whiB7* stress response regulon in Mtb (53). Based on these data, it was hypothesized that *whiB7* upregulation may contribute to acquired drug resistance. Depletion of both *whiB7* and *ettA* in Mtb reversed aminoglycoside resistance. However, *whiB7* knockdown did not reverse ethambutol or levofloxacin resistance, suggesting the mechanism of resistance is *whiB7* independent (53).

Even though *Mycobacterium abscessus* is not closely related to Mtb, it causes debilitating TB-like pulmonary infections and is highly drug resistant, limiting treatment options (90). Understanding *M. abscessus* pathophysiology has been hindered by a lack of genetic tools. Recently, a mycobacterial single-plasmid CRISPRi-dCas9 system optimized for inducible gene silencing in Mtb and *M. smegmatis* was evaluated in *M. abscessus* (56, 91). To test this platform, the authors targeted two well-characterized antimicrobial resistance genes, *bla* and *whiB7*, which are responsible for beta-lactam and macrolide resistance in *M. abscessus*, respectively (92, 93). Depletion of these genes resulted in antimicrobial sensitivity, thereby validating the platform. Following these results, they targeted the essential genes *ftsZ* and *topA* in *M. abscessus*, where the first encodes for the cell division protein FtsZ and the second encodes topoisomerase I. These two genes are also essential in Mtb and *M. smegmatis* and are attractive drug targets (94, 95).

These studies have shown that CRISPRi identification of novel gene pathways involved in multi-drug resistance may be critical to generate successful solutions against the prevalence of MDR Mtb and other mycobacterial strains.

## GRAM-POSITIVE BACTERIA

### Enterococcus faecalis

Native CRISPR loci have been frequently described in *E. faecalis* strains, which hinder foreign DNA acquisition, including resistance plasmids (96, 97). Pathogenic *E. faecalis* strains largely lack functional CRISPR loci, a finding replicated *in vivo* with commensal *E. faecalis* in the mouse intestine (96). One such example is the vancomycin-resistant strain V583, which lost a functional CRISPR system while obtaining antibiotic resistance (97–99). Thus, CRISPRi systems may present an advantageous tool for the examination of MDR in *E. faecalis* clinical isolates. CRISPRi has already been demonstrated in the *E. faecalis* strain OG1RF, an oral infection isolate that possesses one functional native CRISPR system (97). For example, the Kline group developed a multiplex system around streptococcal CRISPRi. They first showed that streptococcal crRNA is not recognized by enterococcal dCas9, preventing interference from the native CRISPR systems of *E. faecalis* strains (15). They then silenced *croR*, which is part of the CroRS two-component system that contributes to antibiotic resistance and intracellular survival within macrophages. *croR* knockdown reduced bacitracin resistance (15), showing similar results to a *croR* transposon-mediated knockout (100).

### Clostridioides difficile

The high prevalence of MDR *Clostridioides difficile* in both community- and hospital-acquired infections has become worrisome due to limited treatment options (101). The frequent use of multiple antibiotics depletes native microfauna in patients’ GI tracts, providing a niche for MDR *C. difficile* to exploit (102). *C. difficile* has species-specific features such as its lipid membrane, which lacks phosphatidylserine and is ~50% glycolipid in composition (57). A titratable CRISPRi system was developed for *C. difficile* to better understand cell wall synthesis, identifying the previously uncharacterized peptidoglycan synthase PBP-0712, which is important for proper elongation, cell division, and protection against lysis (21). Using CRISPRi, it was further confirmed that the two-component system HexRK was essential for antibiotic resistance. This target was initially identified through a transposon insertion library, with a relatively undescribed *B. subtilis* homolog. Silencing of the HexRK operon (*hexSDF*) reduced daptomycin and bacitracin resistance by fourfold and eliminated the unique *C. difficile* glycolipid HNHDRG, which likely contributes to this resistance (57). Silencing of individual HexRK operon genes suggested specific roles for producing HNHDRG, which normally comprises ~16% of the bacterial membrane. HexS and HexD are required for the production of a glycolipid intermediate and its conversion to HNHDRG, respectively, while HexF is not essential (57). As HNHDRG is only found in *C. difficile*, this suggests that CRISPRi silencing of unique features permits deeper dissection of novel pathways, potentially aiding the identification of species-specific druggable targets.

### *Staphylococcus* spp.

*Staphylococcus aureus* is a highly prevalent nosocomial pathogen and possesses resistance strategies against beta-lactams, linezolid, daptomycin, vancomycin, fluoroquinolones, and tetracycline (103). Among the identified mechanisms of antibiotic resistance are efflux pumps and peptidoglycan modification, many of which are the result of direct changes to chromosomal genes via spontaneous mutation or HGT (104). Due to both intrinsic and acquired antimicrobial resistance, treatment of MDR *S. aureus* is extremely challenging (105). To address this challenge, multiple novel CRISPRi systems capable of multi-gene silencing in *S. aureus* have been developed (58, 106).

For example, Zhao and collaborators utilized an anhydrotetracycline (ATc)-inducible promoter to allow inducible and reversible repression of multiple genes. Simultaneous targeting of virulence factors including hemolysin (*hla*) and staphylococcal protein A (*spa*) validated its multi-gene knockdown activity. Using this new system, they were also able to reverse *hla* silencing to baseline expression after removal of ATc. Furthermore, they also silenced *mecA*, a beta-lactam resistance gene in methicillin-resistant *S. aureus* (MRSA) strain N315, sensitizing the MDR strain to oxacillin (58).

Another novel CRISPRi system is pBACi, which successfully silenced a plasmid-bound resistance gene within in a clinical isolate (59). Genetically recalcitrant clinical *S. aureus* isolates possess restriction enzymes that inhibit retention of the conventional vector. To overcome this limitation, pBACi was constructed in B strain *E. coli*, which lacks the components targeted by the *S. aureus* restriction enzymes. Validation of pBACi was done by silencing the β-lactamase gene *blaZ*, which significantly reduced both mRNA levels and β-lactamase activity by ~50% (59).

To perform unbiased whole genome screening, two CRISPRi libraries were created in *S. aureus* strains RN4220 and NCTC8325. The RN4220 library comprised all 2,666 annotated genes and NCTC8325 covered 2,836 genes (20, 60). Screening of the RN4220 library provided evidence that *ctaABM*, *mnhABCDEFG*, and *ndh* are involved in aminoglycoside resistance mechanisms (60). These genes are involved in pathways that lead into the electron transport chain (ETC), which is consistent with the current understanding that disruption of the ETC alters membrane potential and lowers aminoglycoside uptake (60). Additionally, the essential genes in the mevalonate pathway, *mvaD*, *mvaK2*, and Topoisomerase I (*topA*), were also revealed to be novel loci in aminoglycoside sensitivity (60). Furthermore, transcriptional inhibition of several essential ribosomal genes provided gentamicin tolerance, suggesting that ribosomal perturbation may provide some defense against aminoglycosides (60).

The NCTC8325 CRISPRi library was screened against dalbavancin, identifying several genes that confer drug resistance or susceptibility. These genes were involved in cell wall modification (*dltABCD* and *pbp4*), ABC-transporter (*vraFG*), cell division (*ezrA*), ribosomal operon (*rpsF-ssb-rpsR*), and biosynthesis of deoxyribonucleotides (*nrdF*). Additionally, the uncharacterized genes SAOUHSC_00678 and SAOUHSC_00892 (a putative RNA-binding protein) were found to be involved in antimicrobial susceptibility. Interestingly, silencing the lipoprotein KapB, a non-essential gene (*kapB*), only confers resistance to dalbavancin and suggests a unique mechanism of action (20). Finally, this library was used to demonstrate that dalbavancin tolerance is modulated by the metabolic Shikimate pathway, through knockdown of the *sagB*, *aroB*, and *vrfA* genes (20).

Treatment of methicillin-resistant *S. aureus* (MRSA) is often heavily reliant on last-resort antibiotics like vancomycin. When such treatment fails, it is usually due to intermediate vancomycin resistance due to cell wall thickening. By examining RNA-RNA interactions, Mediati and collaborators identified a regulatory region within the 3′UTR of *vigR* mRNA. This *vigR* 3′UTR enhanced *isaA* expression, which is a cell wall lytic transglycosylase (61). Using CRISPRi, *vigR* mRNA expression was reduced, resulting in 1000-fold susceptibility to sub-inhibitory vancomycin concentrations. Knockdown of *isaA* revealed reduction in cell wall thickness, which partially re-sensitized *S. aureus* to vancomycin. This effect was less successful CRISPRi inhibition of *vigR* 3′UTR, which may encourage further CRISPRi use in non-coding genetic regions (61).

## FUNGI

### Saccharomyces cerevisiae

Extending CRISPRi systems to fungal species has faced additional challenges than those present in prokaryotes. For example, diploid fungi pose a barrier in creation of CRISPR-KO strains. Despite such challenges, a plasmid-based, ATc-inducible CRISPRi system was described in the yeast *Saccharomyces cerevisiae*. This yeast CRISPRi system included a dCas9 with the Mxi1 transcriptional repressor fused to the C-terminus of dCas9 (Fig. 1B). This system reduced expression of the targeted gene, and the transcription start site (TSS) and 200 bp upstream of the TSS were identified as the best target site for dCas9 fusion proteins. This technology also uncovered that repression of the C-4 methyl sterol oxidase, Erg25, promoted resistance to fluconazole (23).

### Candida albicans

*Candida* spp. lack a native, autonomously replicating plasmid system and have limited selection targets for plasmid maintenance, presenting an additional barrier to the creation of CRISPRi/a tools. CRISPR-KO tools built around codon-optimized Cas9 were successfully demonstrated in 2015, though essential gene functions were unable to be addressed (107). The first functioning CRISPRi system in *C. albicans* was published in 2019, using two dCas9 fusion constructions, dCas9-Mxi1 and dCas9-Mig1 (22). Both Mxi1 and Mig1 are transcriptional repressors that showed higher silencing efficacy when working together. Contrary to the earlier *S. cerevisiae* system, this CRISPRi construct utilized *C. albicans NEUT5L* homology regions for plasmid integration. Silencing the essential chaperon HSP90 (*ADE2*) validated this system (22). This study showed that like in *S. cerevisiae*, HSP90 also contributed to *C. albicans* fluconazole resistance (22, 108).

Another CRISPR technology recently developed for *C. albicans* allows the modulation of gene expression (109). This system was created by fusing the reporter gene *gfp* to the cytosolic catalase *CAT1* promoter as part of an integrative plasmid containing the CRISPRi system. This initial system maintained sgRNA expression under the constitutive *SNR52* promoter but was modified by utilizing catalytically dead dCas9 under a tetracycline-inducible promoter to allow for both genetic upregulation and downregulation. Its silencing capabilities were validated by targeting *gfp*, reducing expression by ~30%. The silencing capacity was further improved by fusing the hyphal gene repressor Nrg1 to dCas9, reducing *gfp* expression by 45% (109). CRISPR activation (CRISPRa) was developed by exchanging Nrg1 for the transcriptional activator Gal4, showing a twofold increase in *gfp* expression. This was further enhanced by adding a second fusion protein, MCP-V64. The presence of both Gal4 and VP64 transcriptional activators significantly enhanced *gfp* expression (109).

The CRISPRa system was further developed in *C. albicans* to examine antifungal susceptibility (24). This utilized a fusion protein consisting of dCas9 and the tripartite activator complex VPR. Antifungal resistance was examined with the genes *CDR1* and *YAK1. CDR1* is an efflux pump gene associated with azole resistance, and CRISPRa upregulation enhanced transcription by ~2.9- to 5.6-fold resulting in reduced sensitivity to fluconazole. *YAK1* is a kinase whose overexpression promotes filamentation and resistance to the antifungal amphotericin B (110, 111). Using this CRISPRa system, *YAK1* expression was doubled, increasing amphotericin B resistance (24).

### Nakaseomyces glabrata

Formerly considered a member of the *Candida* genus, *Nakaseomyces glabrata* is the second most common cause of candidiasis infection. Clinical isolates have increasingly demonstrated multi-drug resistance, including against azole and echinocandin antifungals (112, 113). Both CRISPRi and CRISPRa have proven useful in the study of *N. glabrata*, taking advantage of dCas9 fusion protein strategies. Using a dCas9-Mxi1 fusion protein, a new CRISPRi system was created and validated via *URA3* silencing (64). Since it was known that *ALG3* deletion in *S. cerevisiae* promotes resistance to the toxin HM-1, this system was tested by silencing the unverified *ALG3* in *N. glabrata*, which proved that reduction of *ALG3* expression promotes fungal growth in presence of the toxin (64, 114).

Recently, CRISPRa utilizing dCas9-VPR has been described in *N. glabrata*. The transcription regulator *PDR1* was first targeted, with 10 unique sgRNAs created due to differences in the reported TSS of *PDR1*. The two sgRNA targeting ~550 bp upstream of the start codon led to a 1.5-fold increase in *PDR1* transcription and subsequent reduction in fluconazole sensitivity (65). This further emphasizes the importance of TSS proximity for successful sgRNA design. This tool was subsequently used to investigate *STE11* and *SLT2* due to their possible roles in drug or environmental resistances. *STE11* mediates tolerance to environmental challenges such as oxidative stress, and *SLT2* is overexpressed when *N. glabrata* is treated with the antifungal caspofungin (115). CRISPRa overexpression of *STE11* was achieved as a 1.2- to 1.8-fold increase and conferred tolerance to H_2_O_2_-induced oxidative stress while *SLT2* was overexpressed 1.8- to 2.8-fold and led to greater *N. glabrata* fitness in the presence of caspofungin (65).

## BIOFILMS

Nosocomial infections frequently involve biofilms on tissue and medical devices (6–8). Biofilms are often associated with antibiotic resistance, promoting chronic and persistent infections, which make them difficult to treat (6–8). Microbial biofilm formation is complex, involving signaling and regulatory pathways that control the transition from motile to sessile lifestyle, metabolic status, cell coordination, production of an extracellular polymeric matrix, and maturation of the biofilm’s 3D structure. Therefore, understanding the intricacies of biofilm formation is critical for developing treatments against infections.

## GRAM-NEGATIVE BACTERIA

### E. coli

*E. coli* biofilms are prevalent on medical devices, contributing to their multi-drug resistance (3, 116). Previous reports have shown efflux pump activity is associated with biofilm formation in *A. baumannii*, *P. aeruginosa*, and *S. enterica* serotype Typhimurium (117–119). CRISPRi targeting of the AcrAB-TolC efflux pump system in *E. coli* inhibited biofilm formation by ~50% in addition to sensitizing to rifampicin, erythromycin, and tetracycline (42). Transcription of *acrA*, *acrB*, and *tolC* was correlated with drug MICs while *acrB* and *tolC* correlated with biofilm formation, though their direct or indirect contributions to biofilm formation remain unclear.

Quorum sensing (QS) is another mechanism important for biofilm formation in *E. coli*. This is facilitated through coordination of cell-cell signaling by producing and detecting autoinducers (AIs) to transcriptionally synchronize behaviors including virulence, biofilm formation, and survival (120–122). Therefore, targeting QS systems may help dissect steps of biofilm formation. The Khan group targeted the *lux*S gene, encoding the synthase of the AI-2 QS molecule, which is critical during the initial stages of biofilm formation (27, 123). This group developed a plasmid-based CRISPRi system to knockdown *lux*S in the *E. coli* clinical strain AK-117. Compared with controls, the CRISPRi-strain exhibited ~95% reduction in *luxS* expression, resulting in ~50% reduction in biofilm formation (27).

### *Pseudomonas* spp.

*Pseudomonas* spp. employ different strategies of environmental persistence including biofilm formation, motility, and production of virulence factors (124, 125). To further understand the mechanisms of *Pseudomonas* spp. pathogenesis and biofilm formation, CRISPRi has been used to elucidate the role of uncharacterized essential genes. For example, the novel, unidentified essential gene PA0715 in the strain PAO1 was targeted by Zhou and collaborators. Its downregulation resulted in reduced growth rate, motility, chemotaxis, antibiotic resistance, pyocyanin production, and biofilm formation (46). To examine the role of PA0715 in virulence, CRISPRi-silenced and control strains were tested in *Galleria mellonella* larvae, finding that downregulation significantly reduced virulence (46). These findings support transcriptomic analyses that showed PA0715 may play a role as a global regulator that influences metabolic signaling pathways affecting motility, biofilm formation, and virulence. While the mechanisms by which PA0715 regulates virulence and biofilm formation are not described, this study paves the way to dissect the role of uncharacterized essential genes.

Additional studies in the non-pathogenic *P. fluorescens* unveiled genes that control biofilm formation. Using three diverse *P. fluorescens* strains, SBW25, WH6, and Pf0-1, Noirot-Gros and collaborators silenced genes involved in the GacA/S two-component system (*gacS*) and regulatory proteins involved in cyclic di-GMP signaling (*bifA*, *dipA*, and *rimA*) (47). They found that silencing of *gacS*, *rimA*, *bifA*, and *dipA* resulted in reduced swarming abilities, affecting biofilm thickness and roughness in varying degrees. The authors suggest that the variability in effects may be due to silencing genes at the beginning of or within operons (47).

## GRAM-POSITIVE BACTERIA

### E. faecalis

Biofilms provide *E. faecalis* a mechanism to colonize inhospitable conditions and, in some cases, include other species in a polymicrobial community. As we previously mentioned, biofilm formation is a very dynamic process, with the potential for genes to become essential at specific stages of development. Afonina and collaborators demonstrated the utility of inducible CRISPRi to study *E. faecalis* genes involved in biofilms in a stage-specific manner (15). The authors targeted the endocarditis- and biofilm-associated pili (Ebp) that plays a key role in *E. faecalis* attachment and biofilm formation (126, 127). By targeting members of the pilus or the whole *ebp*ABC operon, the authors confirmed the importance of the pili in the initial stage of biofilm formation. Furthermore, the role of Ebp pili in biofilm maintenance was tested by silencing the operon after a biofilm formation of 2, 16, or 24 hours, using a nisin-inducible CRISPRi system. All biofilms were incubated for 24 hours post-induction, with Ebp pilus silencing significantly reducing biofilm structure, ultimately indicating that this pilus is important for both biofilm initiation and biofilm maintenance (15). This study shows that nisin could penetrate the entire biofilm and this novel system opens new research venues to investigate the role of conditionally essential genes in a stage-specific biofilm-dependent manner (15).

## FUNGI

### C. albicans

The study of biofilm formation in *C. albicans* has been greatly aided by CRISPRa systems. A dCas9-VPR fusion protein was used to investigate the biofilm-relevant adhesins *ALS3* and *ALS1*. The extent of CRISPRa upregulation of *ALS3* varied with fungal growth conditions. Overexpression of *ALS1* induced robust biofilms; however, excessive upregulation was detrimental to biofilm formation (24). *ALS1* overexpression also enhanced biofilm formation in human urine with fibrinogen, a relevant condition for catheter-associated urinary tract infection (CAUTI) (24). This system was subsequently used in a mouse model of CAUTI, revealing that *ALS1* is critical to biofilm formation and pathogenesis during CAUTI (63).

### N. glabrata

*N. glabrata* makes use of biofilms for pathogenesis, similar to *Candida* species (128). Upregulation of the transcription factor *EFG1* is known to enhance biofilm growth, and this gene has a well-defined TSS. Multiple guide sgRNAs were created, targeting within −462 and +112 bp relative to the TSS. *EFG1* was overexpressed 3.6- to 8.1-fold, and three sgRNAs showed significantly enhanced biofilm forming ability. Each of the three sgRNA targets were within 200 bp upstream of the TSS (−36 bp, −105 bp, and −196 bp), with −105 bp providing the greatest increase in *EFG1* expression. Notably, the CRISPRa construct does repress *EFG1* if the sgRNA targets within the coding sequence. The authors posit that this may be due to steric inference with RNA polymerase II by the dCas9 fusion protein (65).

### Other filamentous fungi

While not all filamentous fungi form complex biofilms, there has been an explosion in the number of filamentous species with developing CRISPRi/a tools in just the last 3 years. In 2021, a CRISPRa vector utilizing a dCas9-VPR transcription activator was described in *Penicillium rubens*. This system targeted the dCas9-VPR complex to the promoter of *macR*, a transcription factor that activates a natively transcriptionally silent gene cluster to initiate macrophorin biosynthesis, an antimicrobial compound (66). In 2022, CRISPRi identified the *Aspergillus niger* gene *DAC1*, a GlcNAc-6-phosphate deacetylase, as controlling spore-like propagule formation, a phenotypic change also associated with drug resistance (67). Finally, in 2023, multiple gene targets within the filamentous fungal rice pathogens *Magnaporthe oryzae* and *Ustilaginoidea virens* were successfully silenced (68).

## OUTLOOK

**Fig 2:**
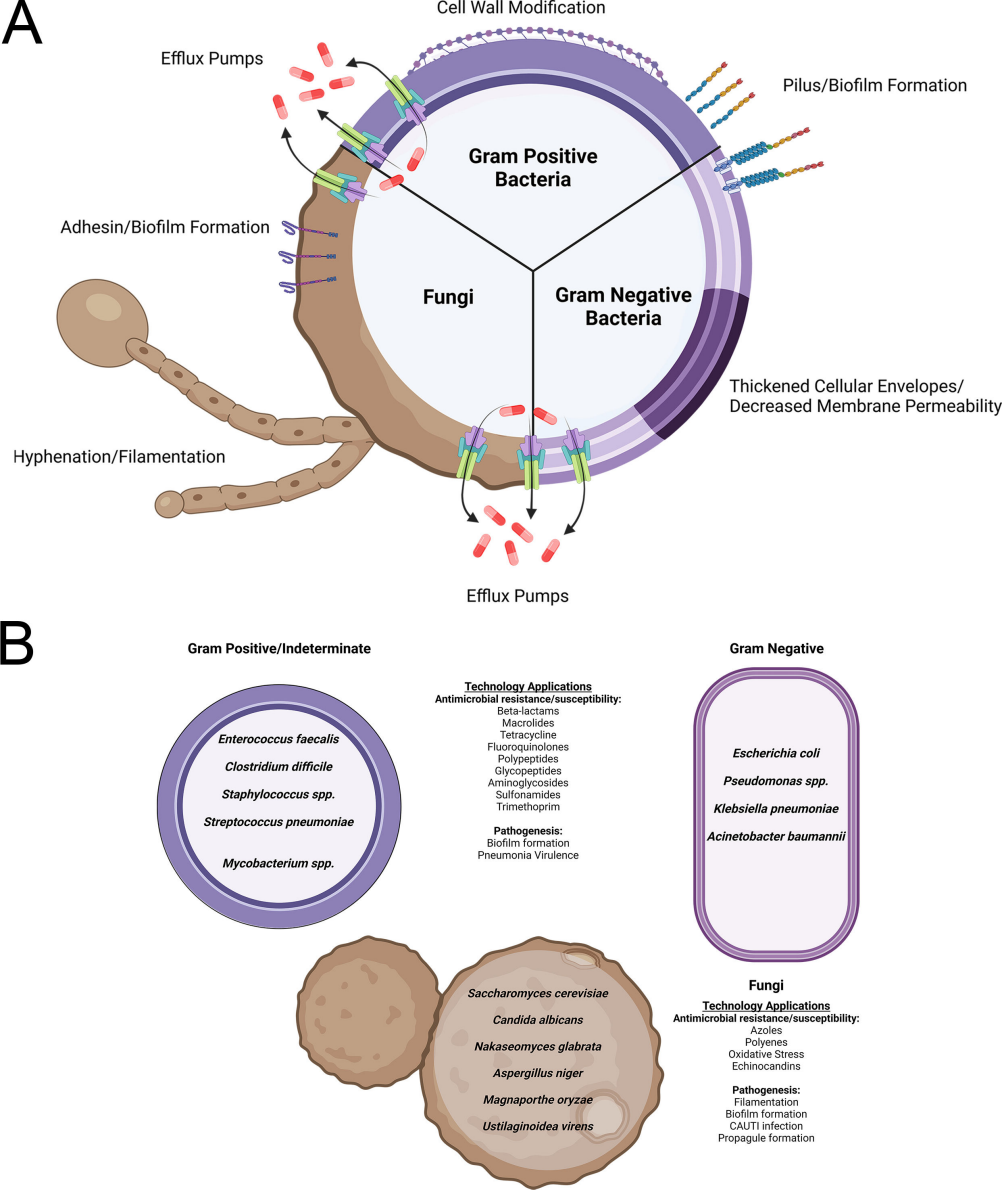
Applications of CRISPRi. Pathogenic microbes have many methods of drug resistance and pathogenic strategies (**A**). CRISPRi has been used across diverse species to investigate these mechanisms. Drug resistance and biofilm formation are particularly represented, given their importance to pathogenesis. CRISPRi/a has also been used successfully to examine other features of fungal pathogenesis (**B**). Figure was created using BioRender.

Nobel-winning work that repurposed the bacterial CRISPR-Cas9 system for genomic editing was described in 2012 (10). Within 1 year, the beginnings of CRISPRi were published utilizing a catalytically dead Cas9 (16, 18). The subsequent decade has seen rapid development of novel gene modulation technologies, with initial applications characterizing essential genes. CRISPRi/a technologies have been developed for a variety of species (Fig. 2B) to better understand mechanisms of MDR and biofilm formation (Fig. 2A).

The use of these gene expression tools in pathogenic species has faced new challenges when compared with genetic editing techniques. The continuous maintenance of CRISPRi systems and design principles for sgRNA have become increasingly sophisticated, as they address two of the greatest challenges to utilizing these technologies.

Retention of CRISPRi platforms must contend with the limited options for selective pressure in highly drug-resistant isolates, as well as native CRISPR systems that target introduced CRISPRi (38, 78). Platforms like pBACi overcome native CRISPRs by including anti-CRISPR proteins, while other tools are built by editing a native system to function as CRISPRi (59, 78). Integrative plasmids can eliminate the need for continual selective pressure; the Mobile-CRISPRi platform takes advantage of this to provide stable gene silencing for over 50 generations in every ESKAPE pathogen (49).

Not all sgRNA sequences are created equal, as the degree of gene silencing will vary by sgRNA sequence. This can be exploited to tune the magnitude of genetic knockdown but can also result in lethal fitness loss (44, 48, 129). Early genome-wide screens in *E. coli* identified specific, five-nucleotide-long sequences within “bad seed” sgRNA that led to toxic, off-target dCas9 activity, exacerbated by high dCas9 expression (44). Some “bad seed” sgRNA sequences have been identified in *E. coli* and proven similarly lethal in other species. However, as some “bad seed” sgRNAs only cause dCas9 toxicity in certain species, this indicates that our knowledge of “bad seed” sequences is incomplete (129, 130).

The risk of ineffective or “bad seed” sgRNA is a constant one for genome-wide screens, especially as most such libraries use 10 or less functional sgRNA per gene. This risk grows as CRISPRi libraries are constructed for less-studied pathogens and isolates, as well as CRISPRi-Seq libraries that benefit from extensive sgRNA coverage of genes. One technology that may prove useful here is CRISPR adaptation-mediated library manufacturing (CALM) (60). CALM adapts a hyperactive Type II CRISPR-Cas machinery for the creation of sgRNA to target a provided genome. CALM was able to generate hundreds of thousands of unique crRNAs for a 95% coverage of all targetable genomic sites in *S. aureus* and manufactured an average of more than 100 unique sgRNA per gene in *E. coli* (60).

CRISPRi/a technologies have also expanded our understanding of essential genes. Genome-wide screens have shown that not all essential genes are vulnerable drug targets nor does *in vitro* essentiality always equate to pathogenic relevance (28, 62). CRISPRi/a shows great promise at identifying genes essential to pathogenesis *in vivo*. CRISPRa in *C. albicans* identified *Als1* as crucial for pathogenesis in a murine CAUTI model (63). CRISPRi has also been used in murine pneumonia models, which identified essential genes to virulence: *exsA* in *P. aeruginosa*; *pal*, *yciS*, and *rib* in *K. pneumoniae*; and *purA* in *Streptococcus pneumoniae* (48, 50, 62). Tailoring promoter strength for constitutive knockdown offers an alternative to induced CRISPRi, alleviating concerns over tissue penetration by some inducing agents (48). Notably Mobile-CRISPRi-Seq showed that the *S. pneumoniae* gene *metK* is dispensable *in vivo*, despite being essential *in vitro (62*). Identifying novel genes and pathways conditionally essential to pathogenesis *in vivo* may offer new insights into drug discovery targets.

CRISPRi/a technologies have revolutionized gene modulation tools over the past decade. The earliest systems developed as Type II dCas9 systems, and these are still utilized as the backbone of many modern tools. In the past few years, new technologies for gene knockdown have been designed utilizing Type I, IV, V, and VI systems (31–33, 37–40). It is likely inevitable that CRISPRa techniques will be expanded to include some of these systems as well, taking advantage of the unique mechanisms across CRISPR types. CRISPRi/a technologies have ushered in a new era of antimicrobial research and are invaluable assets in the investigation of complex pathogenic network interactions across bacterial and fungal species, where multi-drug resistance and biofilm formation still pose significant challenges.
